# The parasitic nematode *Haemonchus contortus* lacks molybdenum cofactor synthesis, leading to sulphite sensitivity and lethality in vitro ^☆^

**DOI:** 10.1016/j.ijpara.2024.11.004

**Published:** 2024-11-29

**Authors:** Robert A. Brinzer, Jennifer R. McIntyre, Collette Britton, Roz Laing

**Affiliations:** School of Biodiversity, One Health and Veterinary Medicine, University of Glasgow, Scotland G61 1QH, UK

**Keywords:** *Haemonchus contortus*, Molybdenum cofactor, Nutrigenetics, Sulphite, Comparative genomics, Nematode

## Abstract

Sulphite oxidase has an essential role in detoxifying environmental and endogenously generated sulphite into sulphate and requires the molybdenum cofactor (Moco) to function. Until recently it was believed that the synthesis pathway for Moco was so important for survival that it was conserved in all multicellular animals. Here we report the use of comparative genomics to identify the absence of the first enzyme involved in Moco synthesis in *Haemonchus contortus*, a highly pathogenic and economically important helminth of livestock that, similar to many parasitic nematode species, has proved difficult to maintain in vitro. We show that Moco deficiency in *Haemonchus* leads to a high sensitivity to environmental sulphite and limits the ability to maintain the early parasitic larval stages in vitro. Analogous losses in Moco synthesis in other recently sequenced nematode species are also identified. These findings may lead to improved culture methods for parasitic nematodes and to novel approaches for their control.

## 1 Introduction

The hematophagous gastrointestinal nematode *Haemonchus contortus* is a highly pathogenic and prevalent parasite of sheep and goats, which can also infect other ruminants of economic value (cattle, deer, antelope and camelids) (Craig, 1993; Tapia-Escárate et al., 2015; Abbas and Hildreth, 2022; Irshad et al., 2023; Khan et al., 2023; Zahid et al., 2023). Annual global economic losses caused by this parasite are estimated to exceed $1.3 billion in production losses and treatment costs (Waller and Chandrawathani, 2005; Qamar et al., 2011; Emery et al., 2016; Mavrot, F., 2016. Live-stock nematode infection in a changing world: investigating the European situation. Doctoral dissertation, Vetsuisse Faculty, University of Zürich, Switzerland. https://doi.org/10.5167/uzh-125799). Despite being highly successful in host species, parasitic stages of *H. contortus* are unable to be reared under laboratory conditions for more than a few days before dying despite numerous in vitro cultivation attempts which began almost a century ago (Lapage, 1933). The inability to rear consecutive generations of *H. contortus* under controlled conditions means there is a requirement for infection of animal hosts. This increases experimental variability, has associated costs and ethical concerns, and severely limits the application of molecular genetic techniques to study gene function in parasitic life stages. This contrasts with the model organism, and fellow clade V nematode, *Caenorhabditis elegans*, which can be reared indefinitely on chemically-defined media (Szewczyk et al., 2003).

Nutrigenetics is the study of how the genome influences nutritional requirements, especially in relation to avoiding malnutrition-related disease states (Otero and Bernolo, 2023). This means that comparative genomics can be used to predict nutrient requirement differences between populations or species based on non-redundant gaps in metabolic pathways that are present in one genome and not the other (Seif et al., 2020; Otero and Bernolo, 2023). With an increasing number of nematode genomes available, it allows for comparison between different clades or genera for genes and metabolic pathways that may have been lost or gained (Coghlan et al., 2019). In this study, the genomes of *H. contortus* and *Caenorhabditis elegans* were compared to probe the possible underlying causes of the differences in the ability to adapt to laboratory culture. We find that *H. contortus* lacks genes in a number of pathways including responses to stress, phospholipid metabolism and osmolyte synthesis. Here we also report a gap in the first step of the molybdenum cofactor (Moco) synthesis pathway and show that it causes Moco deficiency in this species in the absence of a dietary source. This results in a sensitivity to sulphite from both exogenous and endogenous sources that limit in vitro cultivation. A comparative genomics study of the other 136 nematode species with available genomes (WormBase ParaSite release 18) identified potentially identical auxotrophies in other parasitic nematode species. This approach can potentially help uncover required supplements for nematode in vitro culture and highlight novel targets for parasite control strategies.

## 2 Materials and methods

### 2.1 Comparative genomics

The *H. contortus* (PRJEB506, MHCO3ISE_4.0; Doyle et al., 2020) and *C. elegans* (PRJNA13758, WBCEL235; Harris et al., 2013) proteomes were compared using OrthoFinder v2.5.4 (Emms and Kelly, 2019) to generate lists of proteins which were shared or only present in one of the species. From these lists, proteins were annotated using the Kyoto Encyclopedia of Genes and Genomes (KEGG) Automatic Annotation Server (KAAS; https://www.genome.jp/kegg/kaas/ (accessed March 2023); Moriya et al., 2007) and visualised on KEGG Mapper v5.1 (Kanehisa et al., 2022). In KAAS, *H. contortus* peptide sequences were identified using a bi-directional Protein-protein Basic Local Alignment Search Tool (BLASTP) including 30 different species. The *C. elegans* peptide sequences were identified using *C. elegans* data present within KAAS and a single directional BLASTP. Details of the scripts run and parameters used can be found at https://github.com/SheepwormJM/Orthofinder-analysis-for-Haemonchus-contortus-vs-Caenorrhabditis-elegans-Brinzer-et-al-2024 (last accessed 26 March 2024) and in the generated log file (Supplementary Data S1).

### 2.2 Haemonchus strain, egg isolation, bleaching and synchronization

Provision of *H. contortus* materials was covered by UK Home Office license number **PP6939295** and the Moredun Research Institute Experiments and Ethics Committee, UK, ethical approval numbers **MRI E27/19, MRI E02/22** and **MRI E05/23**.

The MHco3(ISE) (Moredun *Haemonchus contortus* isolate 3: Inbred Susceptible Edinburgh; Roos et al., 2004) strain was maintained at the Moredun Research Institute (Scotland, UK). Eggs were separated from faecal matter using a standard salt floatation technique (Stoll, 1930) and surface sterilized by bleaching for 5 min (0.25 M KOH (Fisher Scientific, UK: #P/5640/53) and 10% v/v hypochlorite solution (Merck, UK: #1.05614)) before washing three times with excess M9 buffer (3 g of KH_2_PO_4_ (Scientific Laboratory Supplies, UK: #CHE2950), 6 g of Na_2_HPO_4_ (Sigma Aldrich, UK: #S9763), 5 g of NaCl (VWR Chemicals, UK: #27810.295) and 1 mM of MgSO_4_ (VWR Chemicals: #291184P) per litre) and centrifugation for 1 min at 1150 *g*. The eggs were then transferred to a 5 cm petri dish and the volume brought up to 5.5 ml using M9 before hatching overnight at 26 °C. L1s were quantified by counting the number in 10 μl pipetted onto a glass slide, after thorough agitation, using a Stemi 2000 microscope (Zeiss, Germany).

Adult ISE *H. contortus* were provided by the Moredun Research Institute from experimentally infected sheep that had been euthanized 42 days p.i. according to the methods of Doyle et al., 2022. Adult worms were rinsed from the abomasal surface with warm physiological saline and maintained at 37 °C while sexed and picked into 37 °C RPMI-1640 (Fisher Scientific: #31870) before immediate use.

### 2.3 Caenorhabditis elegans maintenance and bleaching

The *C. elegans* N2 strain was maintained according to standard procedures (https://www.wormbook.org/toc_wormmethods.html) (last accessed 26 March 2024; Stiernagle, 2006) on nematode growth medium (NGM) plates inoculated with a lawn of OP50-1 *E. coli*. Worms were synchronized by bleaching young adults for 8 min to release and axenize eggs which were then washed and treated as described for *H. contortus* (Section 2.2), except that eggs were hatched at 21 °C.

### 2.4 Sulphite exposure assays

A fresh 2.5 M sodium metabisulphite (Sigma Aldrich: #S900) stock solution was made by dissolving 0.4753 g in 1 ml of 1.4 M NaOH (Fisher Scientific: #S/4920/53), final pH 6.5. Next, 990 μl of nutritive media (10 g of yeast extract (Oxoid, UK: #LP0021), 8.1 g of NaCl (VWR Chemicals: #27810.295) and 100 ml of Earle’s Balanced Salt Solution with carbonate buffer (Sigma Aldrich: #H8264-500ML) per litre) were inoculated with 10 μl of OP50-1 *E. coli* culture (in Luria-Bertani (LB) Broth with 50 μg/ml of streptomycin (Fisher Scientific: #11860038)), mixed and split into two 500 μl aliquots. To one of the aliquots 1.6 μl of sodium metabisulphite stock solution was added and mixed to make an 8 mM solution. Varying ratios of the two nutritive media were then mixed to generate a sodium metabisulphite dose range between 0 mM and 8 mM in 0.5 mM increments. The sodium metabisulphite stock was diluted in distilled water and added to the wells of 96 well plates (Corning Incorporated, USA: #351172; separate plate per dose) before adding 200 μl of molten 1.5% w/v agar (Sigma Aldrich: #05038) in distilled water to produce a series of 0.5 mM increments. To the agar-filled wells, 30–50 L1s of either *H. contortus* or *C. elegans* were pipetted before adding 50 μl of sodium metabisulphite containing nutritive media with the same concentration as the agar. Empty wells on the opposite side of the plate were filled with distilled water to maintain the humidity and the plate was sealed with parafilm before counting the initial worm numbers. Plates were subsequently incubated at 26 °C in a sealed plastic box. Survival and development were recorded every day for 8 days after setting up the experiment. Assays were performed in triplicate. Data was processed in Microsoft Excel according to a standard dose response pipeline by converting the number of responders (developmental delay or death) into a percentage, correcting for control mortality using the Schneider-Orelli variant of the Abbott’s formula (Schneider-Orelli, 1947), using a Grubbs’ test to check for outliers (Grubbs, 1969) and converting the data into a linear model using the probit transform (Bliss, 1934) before determining the IC_50_/LC_50_ (half-maximal inhibitory concentration/50% lethal concentration) and associated 95% confidence intervals (CIs) with linear regression.

Exposure of adult worms to sulphite was carried out by adding 2 μl of sodium metabisulphite stock solution to 1 ml of RPMI-1640 (Sigma Aldrich: #6504) reconstituted with 20% v/v FCS (Gibco, USA: #10082147), 25 mM HEPES (Gibco: #15630056), 2 g/L of sodium carbonate (Sigma Aldrich: #451614), 60 μg/ml of penicillin (Fisher Scientific: #15140–122) and 100 μg/ml of streptomycin (Fisher Scientific: #15140–122), pH 7.4 (5 mM sodium metabisulphite content) before transferring 500 μl aliquots into two wells of a 24 well tissue culture plate (Corning Incorporated: #3473). Two control wells of the RPMI formulation without sulphite were also added before filling the remaining unoccupied wells with 1 ml of sterile distilled water to maintain the humidity. To each RPMI-containing well, five adult *H. contortus* males or females were transferred using a porcupine quill and incubated at 39 °C with a 10% CO_2_ atmosphere. Assays were performed in triplicate for males and females, and mortality was scored, based on complete lack of movement over a 30 s period, absence of pharyngeal pumping, unresponsiveness to tapping the wells or touch and rod-like appearance (rigor mortis), at 24 h and 48 h after setup. Images were taken using a Leica DM IL microscope at 40x magnification with a CM-8 Universal Phone Adapter (Eyeskey Optics, USA) for mobile phone cameras and a Hauwei Honor 6x or Apple iPhone 12 mini mobile phone.

### 2.5 Moco depletion assays

Moco depletion in *H. contortus* using a ΔMoaA bacterial strain was tested both on agar plates and in liquid culture.

NGM plates were inoculated with either OP50-1 (produces Moco) or Moco-deficient ΔMoaA bacteria (JW0764-KC; NBRP, Keio Collection; Baba et al., 2006). ΔMoaA was grown in LB broth with 50 μg/ml of kanamycin (Sigma Aldrich: #60615). The bacterial lawns were positioned opposite where approximately 300*H. contortus* L1s were subsequently pipetted. Larvae were left overnight to migrate onto the bacterial lawn before using a sterile scalpel to remove a section of the NGM agar around where the larvae were first deposited to eliminate any residual contamination that might have survived the bleaching. The developmental stages and number of dead worms were recorded daily for 8 days.

Well format assays for controls were set up similar to the control wells for the sulphite assay (Section 2.4) while the experimental group used nutritive media that had been inoculated with ΔMoaA instead of OP50-1. Survival and development were monitored daily for a minimum of 8 days.

All assays were performed in triplicate and differences in mortality were compared using a Student’s *t*-test.

### 2.6 Sulphite oxidase (SUOX) activity assay

SUOX assays were carried out according to the method of Oliphant et al. (2023) using a timepoint of 4 days which was selected based on the transition of the larval population from L2s into non-feeding L3s. In short, ~3000 L1s of either *H. contortus* or *C. elegans* were pipetted onto NGM plates with 300 μl of OP50-1 or ΔMoaA lawns and allowed to grow for 96 h before collection by washing off the plates with M9. The worms were washed three times in M9, with 1150 *g* centrifugations for 3 min, before being left in excess M9 on a rocker for 1 h to clear gut contents, before a fourth wash and centrifugation. Worms were transferred to an Eppendorf tube and pelleted at 10,000 *g*, and excess supernatant removed using a pipette. To release sulphite oxidase, the pellet was frozen in liquid nitrogen twice while homogenizing in 50 μl of lysis buffer (20 mM HEPES (Gibco: #15630056), 150 mM NaCl (VWR Chemicals, UK: #27810.295), 1 mM EDTA (Sigma Aldrich: #E4884), 0.5% Triton X-100 (Sigma Aldrich: #T8532-500ML) and adjusted with 2 ml/L of 5 M NaOH (Fisher Scientific: #S/4920/53) to pH 7.5) using an Eppendorf pellet pestle. Samples had an extra 350 μl of lysis buffer added, were briefly vortexed and then incubated on ice for 30 min before protein quantification using a Pierce bicinchoninic acid (BCA) Protein Assay kit (Thermo Fisher Scientific: #23225) according to the manufacturer’s instructions.

Activity buffer (100 mM Tris-HCl (Fisher Scientific: #BP1521), 0.1 mM EDTA (Sigma Aldrich: #E4884), 0.04 mM cytochrome C_ox_ and pH 8.5) was prepared as a 1.053x stock lacking cytochrome *C* and using 50 ml/L of 1 M HCl to adjust the pH to 8.5. For each sample the activity buffer was reconstituted at least 4 h before starting the assay by mixing 1045 μl of buffer stock with 55 μl of 0.8 mM cytochrome *C* stock (Sigma Aldrich: #C3131-10MG, dissolved in distilled water and stored as single use aliquots at −20 °C) and periodically vortexed while incubated on ice, to allow the cytochrome *C* to fully oxidise, until use. Samples of worm homogenate were diluted to 10 μg of protein per 50 μl in lysis buffer or used undiluted. Assays were carried out in 96 well clear bottom plates (NUNC, USA: #265302) by adding 180 μl of activity buffer to 50 μl of sample and set up as two rows in triplicate. The plate was incubated on ice for 5 min before being transferred to the proximity of the plate reader. To the wells, 20 μl of distilled water or freshly prepared 5 mM sodium sulphite (Sigma Aldrich: #S0505-250G) were added before immediately inserting the plate into the plate reader (BMG Labtech, Germany: PHERAstar FS). The plate was shaken using orbital movement at 200 rpm for 5 s before measuring absorbance at 550 nm in kinetic mode for 720 cycles with a 6 s cycle time and 10 flashes per well. Path length correction was enabled with 2 mm orbital averaging.

All reagents were treated using sterile technique to avoid false positives caused by bacterial contamination. Data was processed by subtracting the average absorbance value of the water-treated wells from the sodium sulphite-treated wells at each time point before plotting the difference against time in Microsoft Excel. The gradient of the trendline for the linear portion of the graph was then used to determine the activity of SUOX in units (U) (lmol/ min) (16.6667nkat) per mg of protein in the homogenate. The SUOX activity of different experimental groups was then compared using a Student’s *t*-test.

### 2.7 Haemonchus contortus L4 in vitro culture

Approximately 3000 axenized *H. contortus* L1s were reared on NGM plates that had been seeded with OP50-1 or ΔMoaA and treated as described for the Moco depletion plate assays (Section 2.5). The plates were incubated for 120 h to ensure the entire population had developed into infective L3s before harvest by washing. The worms were then centrifuged at 1150 *g* for 1 min and washed once with sterile distilled water before centrifuging at 3000 *g* for 30 s and removing as much supernatant as possible. The tube was made up to 900 μl using M9 before adding 100 μl of Milton Sterilizing Fluid (2% hypochlorite solution; Milton International, UK) and briefly mixed before observation under a light microscope until 50% exsheathment could be observed (typically occurred within 1 min of incubation). Worms were pelleted by centrifugation at 1150 *g* for 1 min and washed with M9 three times before transfer to a new tube. Two additional washes in M9 with 60 μg/ ml of penicillin (Fisher Scientific: #15140–122) and 100 μg/ml of streptomycin (Fisher Scientific: #15140–122) were carried out before a final wash and resuspension in Earle’s Balanced Salt Solution (Sigma Aldrich: #H8264-500ML) (pH5) with 60 μg/ml of penicillin (Fisher Scientific: #15140–122) and 100 μg/ml of streptomycin (Fisher Scientific: #15140–122). To each well of a 24 well tissue culture plate, ~300 exsheathed *H. contortus* L3s were transferred, with the volume made up to 250 μl with Earle’s Balanced Salt Solution (Sigma Aldrich: #H8264-500ML) (pH5).

The plate was then incubated under humid conditions at 39 °C and a 10% CO_2_ atmosphere for 72 h before adding 250 μl of RPMI-1640 (Sigma Aldrich: #6504) reconstituted with 20% v/v FCS (Gibco, USA: #10082147), 25 mM HEPES (Gibco: #15630056), 2 g/L of sodium carbonate (Sigma Aldrich: #451614), 60 μg/ml of penicillin (Fisher Scientific: #15140–122) and 100 μg/ml of streptomycin (Fisher Scientific: #15140–122), pH 7.4. Mortality was then scored daily until all worms were deceased. Data was processed in Microsoft Excel to generate Kaplan-Meier curves which were then compared with a Log Rank Test.

### 2.8 Nematode genome BLAST search

The amino acid sequences of *C. elegans* MOC-1, MOC-2, MOC-3, MOC-4, MOC-5, MOC-6 and F49H6.5 were obtained from Worm-Base (https://wormbase.org// (last accessed 26 March 2024)). Human MOCS2A from UniProt (https://www.uniprot.org/unipro-tkb/O96033/entry (last accessed 26 March 2024)) and *Brugia malayi* Bm10818 from WormBase ParaSite (https://parasite.worm-base.org/index.html (last accessed 26 March 2024)) were selected as distant MOC-6 orthologs. Sequences were entered into the BLAST Sequence Search tool of WormBase ParaSite. A protein query was used against all Nematoda for both protein and DNA databases using the BLASTP and TBLASTN search tools, respectively, with the “Maximum number of alignments displayed” and “Maximum number of scores displayed” set to 1000. The list of resulting genomes was then manually subtracted from the list of input genomes listed under the job details. A reciprocal BLAST search was performed on each hit to avoid false positives.

## 3 Results

### 3.1 Comparative genomics reveals metabolic capabilities that have been gained and lost in H. contortus

OrthoFinder identified 10,118 shared/orthologous genes in the *H. contortus* and *C. elegans* genomes. *Haemonchus contortus* lacked an ortholog for 9,460 genes but encoded 9,503 which were not present in *C. elegans*. Of these genes, KAAS applied functional annotation to 5,212 (51.5%) of the shared genes, 1,067 (11.3%) of the genes not found in *H. contortus* but present in *C. elegans* and 586 (6.2%) of the genes not found in *C. elegans* but present in *H. contortus* (Supplementary Table S1). Of the functionally annotated genes unique to each species, 33 (5.6%) of *H. contortus* and 648 (60.7%) of *C. elegans* genes were chemoreceptors. Within the data set, it was observed that *H. contortus* has gained genes with roles in protein degradation, chromatin remodelling and transcriptional regulation, DNA repair and replication, RNA splicing and translation, neural development, vertebrate immune system modulation, peptidase inhibition, vitamin K recycling, methylglyoxal metabolism, transport of sugars, sterols, nucleotides and amino acids, NADH metabolism and carbon dioxide assimilation (Supplementary Table S1). The OrthoFinder data also showed a large divergence in the expansion and loss of protein family members involved in fatty acid metabolism, protein glycan synthesis, phospholipid head recycling, IP_3_ and WNT signalling, and chemoreception pathways between the two species (Supplementary Table S1). Compared with *C. elegans, H. contortus* lacks genes with functions involved in cell cycle and apoptosis regulation, meiotic crossover and double strand break repair, head and vulval morphology, osmolyte synthesis and osmotic regulation, transporters for RNA, long peptides and sphingolipids, peptide palmitoylation, the catabolism of sphingosine, ceramide and cysteine, dauer regulation, metal toxicity tolerance, behavioural responses to starvation, hypoxia and heat stress, and molybdenum cofactor synthesis (Supplementary Table S1).

### 3.2 Haemonchus contortus is sensitive to exogenous sulphite

**Fig. 1:**
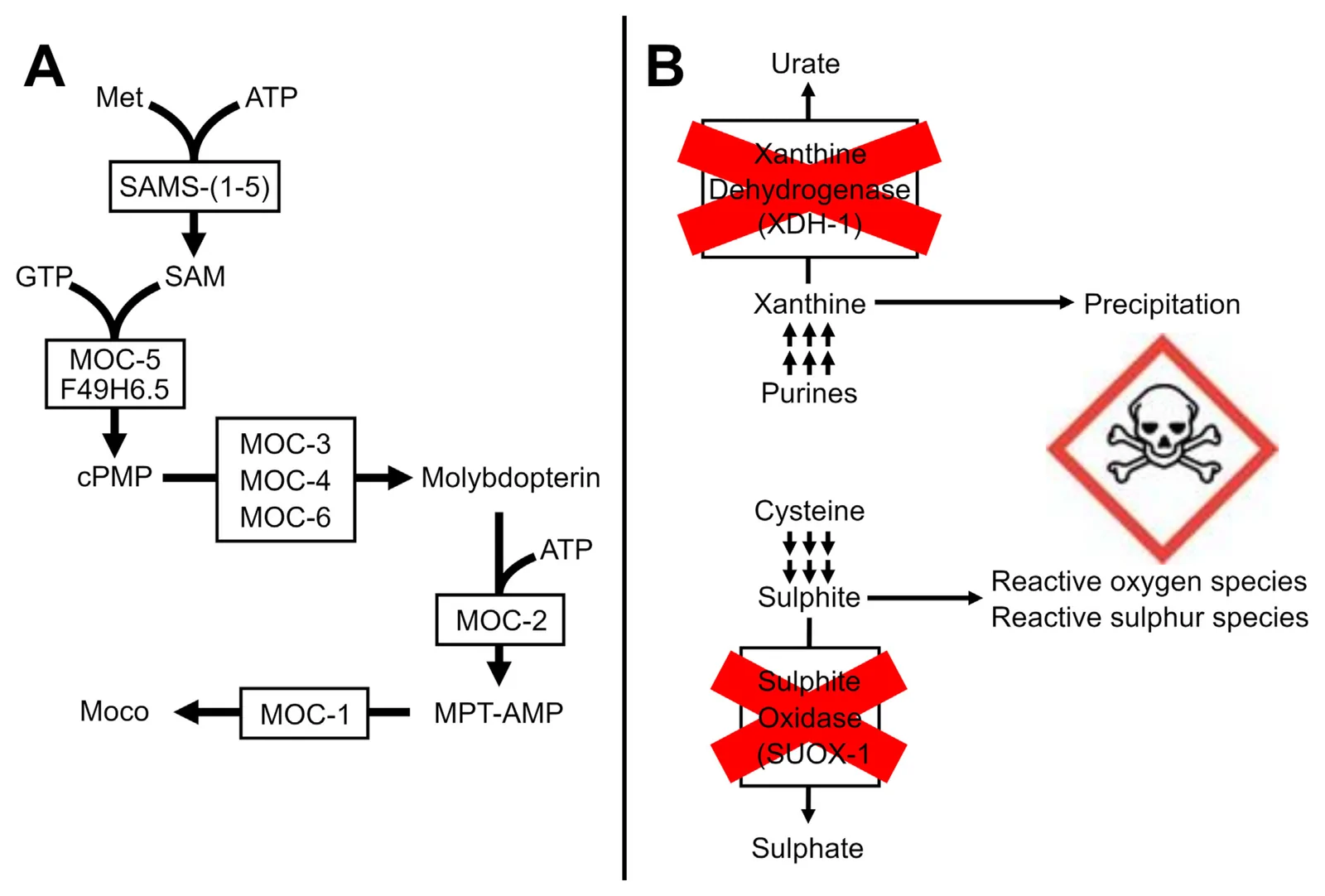
Molybdenum cofactor synthesis pathway and consequences of deficiency in nematodes. (A) Molybdenum cofactor (Moco) synthesis pathway in Caenorhabditis elegans. Boxes, enzymes with subunits listed within; Met, methionine; SAM, S-adenosyl methionine; cPMP, cyclic pyranopterin monophosphate; MPT-AMP, adenylated molybdopterin; Moco, molybdenum cofactor. Gene names used are C. elegans orthologs. (B) Consequences of Moco deficiency (Giovannuzzi, 2024). Crosses, loss of enzyme function due to a lack of Moco.

As a predicted incapacity to synthesize molybdenum cofactor is unusual, this pathway (Fig. 1A) was chosen for further investigation. Lack of Moco is associated with pathology caused by accumulations of xanthine, and more importantly sulphite (Fig. 1B). *Caenorhabditis elegans* mutants in the Moco synthesis pathway have been characterized as showing sulphite sensitivity, caused by reduced sulphite oxidase activity (Warnhoff and Ruvkun, 2019; Oliphant et al., 2023). This results in early developmental arrest and lethality without a dietary source of Moco, or exacerbates developmental delays during sulphite exposure if a dietary source of Moco is available, compared with the wild type (Warnhoff and Ruvkun, 2019; Oliphant et al., 2023). The comparative genomics analysis indicated that *H. contortus* lacks orthologs of *moc-5, f49h6.5* and *moc-6*, which are all necessary for Moco biosynthesis (Fig. 1A). Therefore, impacts of sulphite on *H. contortus* development, and the absence of dietary Moco on sulphite oxidase activity and survival, were probed.

When the early free-living stages (starting at L1) of *H. contortus* were exposed to sodium metabisulphite, mortality was observed beginning at 1 mM with 100% mortality at doses above 4 mM (Supplementary Fig. S1A). Most mortality occurred within 4 days of starting the experiment (Supplementary Fig. S1B) after which the calculated LC_50_ was found to remain relatively constant (Supplementary Fig. S1C). On day 6, the standard timepoint for most *H. contortus* L1s in larval development assays to have developed to L3s (Crook et al., 2016), the LC_50_ value was 2.51 mM (95% CI = 1.91–3.3 mM)(*N* = 2,165). As the observed phenotype differed from the non-lethal developmental delay reported for *C. elegans* N2s exposed to 5 mM sodium metabisulphite (Oliphant et al., 2023), a control experiment using *C. elegans* with a dose range of 1– 4 mM sodium metabisulphite (*N* = 2,290) was carried out to account for differences in methodology. No significant mortality was observed in *C. elegans* N2s at the highest dose compared with the control before completion of the lifecycle and food source depletion, while the reported developmental delay was noted at doses above 1.5 mM (data not shown).

**Fig. 2:**
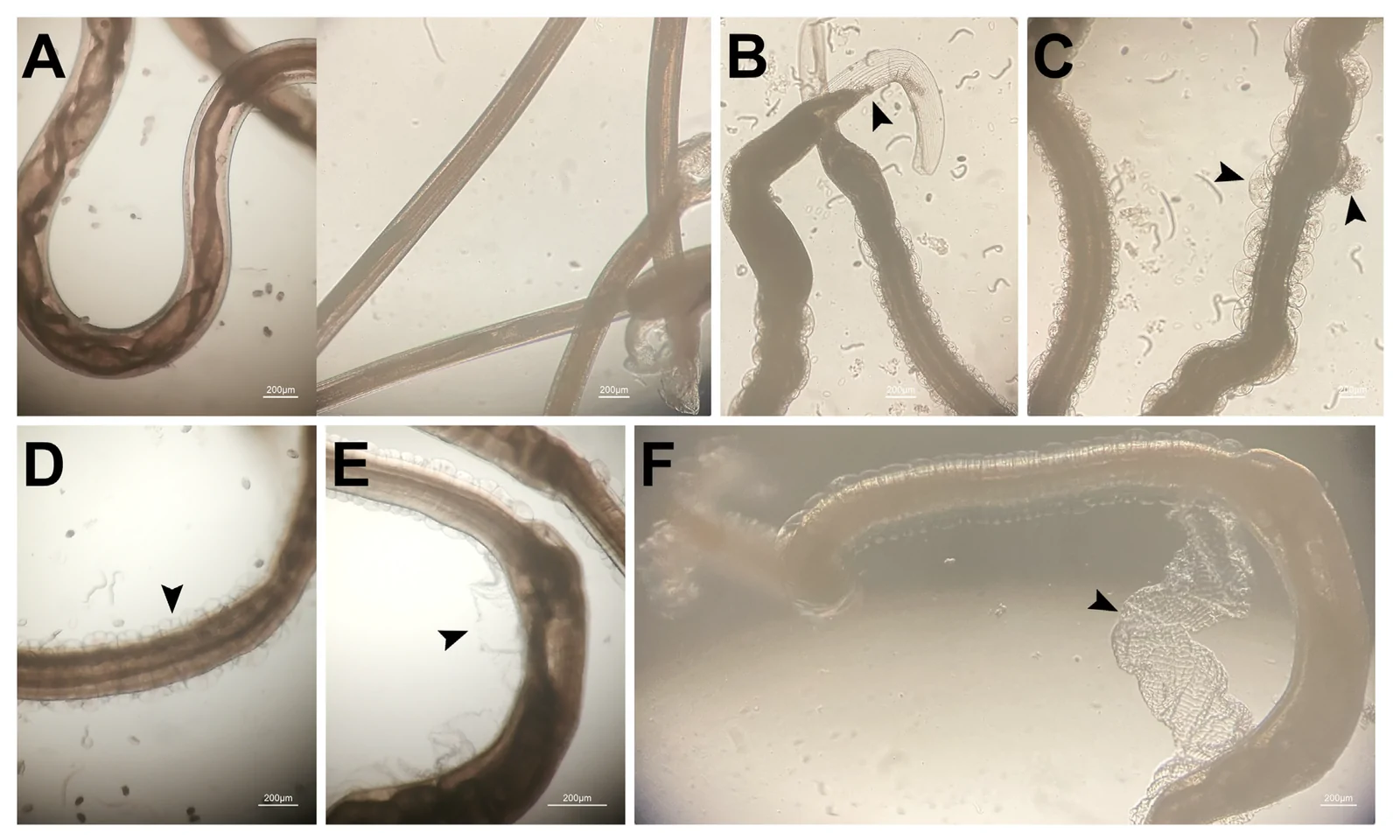
Cuticle damage caused by sulphite exposure in adult *Haemonchus contortus*. (A) Controls showing no cuticle damage; representative female on the left and male on the right. (B) Females, still living, showing detachment and slippage of the cuticle from the mouth (foreground) and blistering of the cuticle (background). (C and D) Additional images of females showing blistering of the cuticle. (E) Female, dead, showing breakage of the cuticle. (F) Male, dead, showing detachment of damaged cuticle. Black arrows are to draw attention to areas of cuticle showing substantial damage. Images were taken at 40x magnification.

The susceptibility of the adult parasitic stages of *H. contortus* to sulphite was also investigated. As the adults can only be cultivated in vitro for a few days, a 5 mM sodium metabisulphite dose was chosen as it caused 100% mortality within 48 h for the L1 stage (Supplementary Fig. S1D). Significant mortality was observed in both males (100%, *N* = 15, *P* = 0) and females (93.3 ± 11.5%, *N* = 15, *P* = 0.005) after 24 h of exposure and 100% mortality at 48 h. During this timeframe there was no death among the male controls, cultured in the absence of sulphite, while female controls showed 0% mortality at 24 h and 13.3 ± 11.5% by 48 h. It was noted that unlike the *H. contortus* L1 to L3 free living stages, which died without additional observable phenotypes, the cuticle of adults showed blistering, detachment, slippage and disintegration upon sulphite exposure (Fig. 2B-F).

### 3.3 Dietary Moco is required for H. contortus larval survival and sulphite oxidase activity

**Fig. 3:**
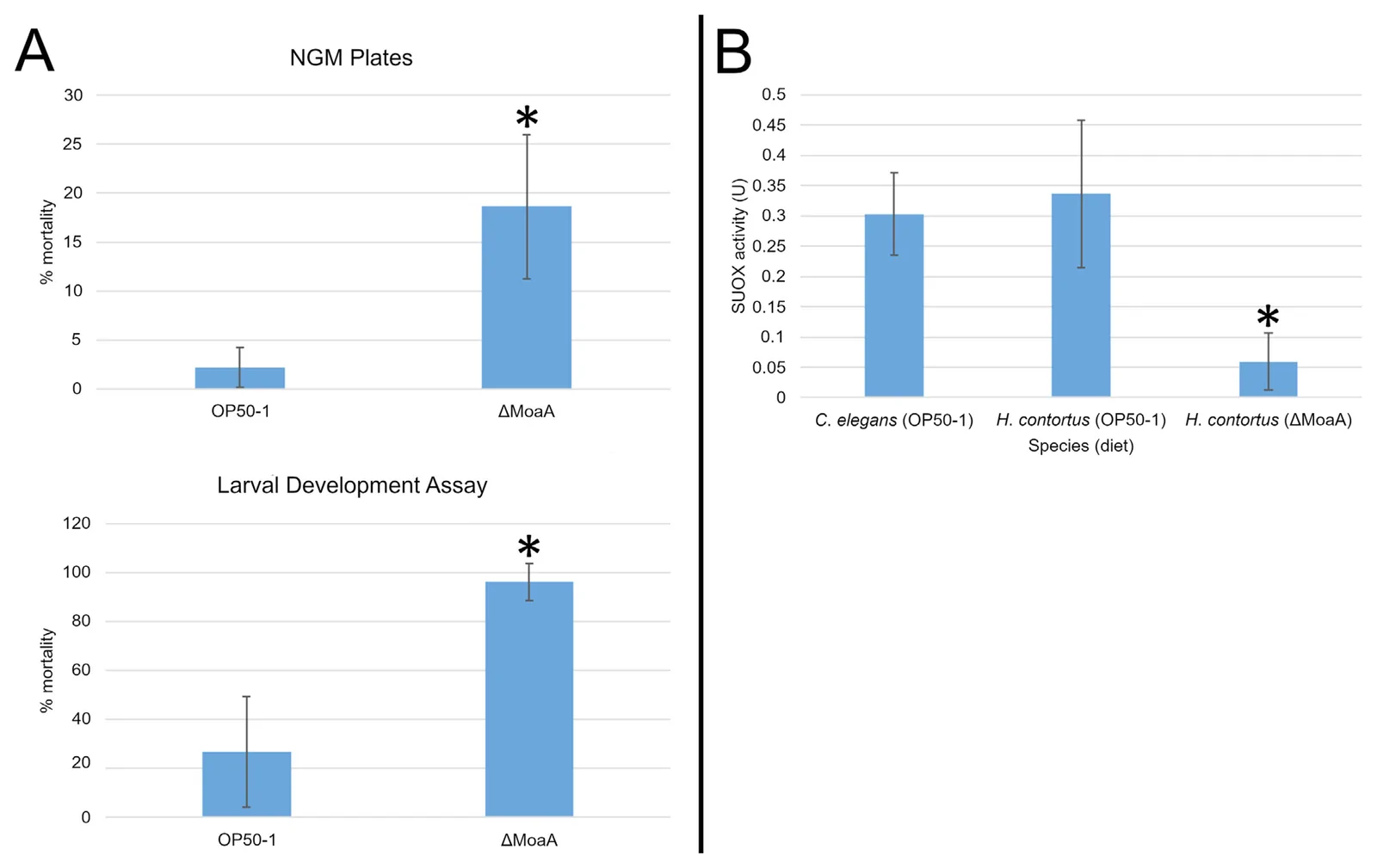
Mortality and sulphite oxidase activity when *Haemonchus contortus* is fed a Molybdenum cofactor (Moco)-depleted diet. (A) Mortalities of *H. contortus* free-living larvae when fed a Moco-containing diet (OP50-1) and Moco-deficient diet (ΔMoaA) on nematode growth medium (NGM) plate or larval development assay format systems. *N* = 883 (OP50-1, NGM plate), 990 (ΔMoaA, NGM plate), 172 (OP50-1, larval development assay) and 241 (ΔMoaA, larval development assay). *P < 0.05. (B) Sulphite oxidase activity in *H. contortus* infective L3s (iL3s) after being fed on a Moco-containing OP50-1 diet and Moco-deficient ΔMoaA diet. Wild type *Caenorhabditis elegans* N2 was used as a positive control for the methodology.

Rearing the free-living stages of *H. contortus* monoxenically on NGM plates with *E. coli* lacking Moco and synthetic intermediates (ΔMoaA bacteria) caused no significant mortality in the L1 and early L2 developmental stages. However, at the L2-L3 transition a proportion of the population showed lethargy and death. Larvae reared on NGM control plates with OP50-1 had 2.2 ± 2% mortality (*N* = 883) compared with 18.6 ± 7.4% mortality on the Mocodeficient bacteria plates (*N* = 990) (Fig. 3A). Additionally, *H. contortus* L1 to L3 development was examined in liquid culture where there is less exposure to air. Notably, those reared in the wells with liquid medium had 26.6 ± 22.4% mortality for controls (*N* = 172) and 96.1 ± 7.6% mortality without dietary Moco (*N* = 241) (Fig. 3A). This represents a significant increase in mortality on both the NGM plate (*P* = 0.014) and submerged liquid medium (*P* = 3.32*10^-5^) formats.

To verify that lack of a dietary source of Moco was causing progressive Moco depletion with a consequential reduction in sulphite oxidase activity, sulphite oxidase assays were carried out on crude homogenates using *C. elegans* samples as a positive control for establishing the assay. Sulphite oxidase activity in wild type *C. elegans* N2 fed on a Moco-deficient diet was not investigated as long-term depletion is already known to cause a reduction comparable with short-term depletion in Moco synthesis mutants (Oliphant et al., 2023). *Caenorhabditis elegans* fed on OP50-1 had sulphite oxidase activity of 0.303 ± 0.068 U/mg of protein (5.05 ± 1.13 nkat/mg) and *H. contortus* had 0.336 ± 0.121 U/mg of protein (5.6 ± 2.02 nkat/mg) when fed on OP50-1 but only 0.06 ± 0.047 U/mg of protein (1 ± 0.78 nkat/mg) on the Moco-deficient diet (Fig. 3B). This means that without a dietary source of Moco, the capacity of *H. contortus* to detoxify sulphite is significantly reduced (*P* = 0.005) but some residual sulphide oxidase activity persists even after 4 days of Moco deficiency. Later time points were not investigated as L3s would have stopped feeding and become dependent on depleting endogenous reserves until reaching the L4 stage so decrementing sulphite oxidase activity would be expected in both treatment groups.

**Fig. 4:**
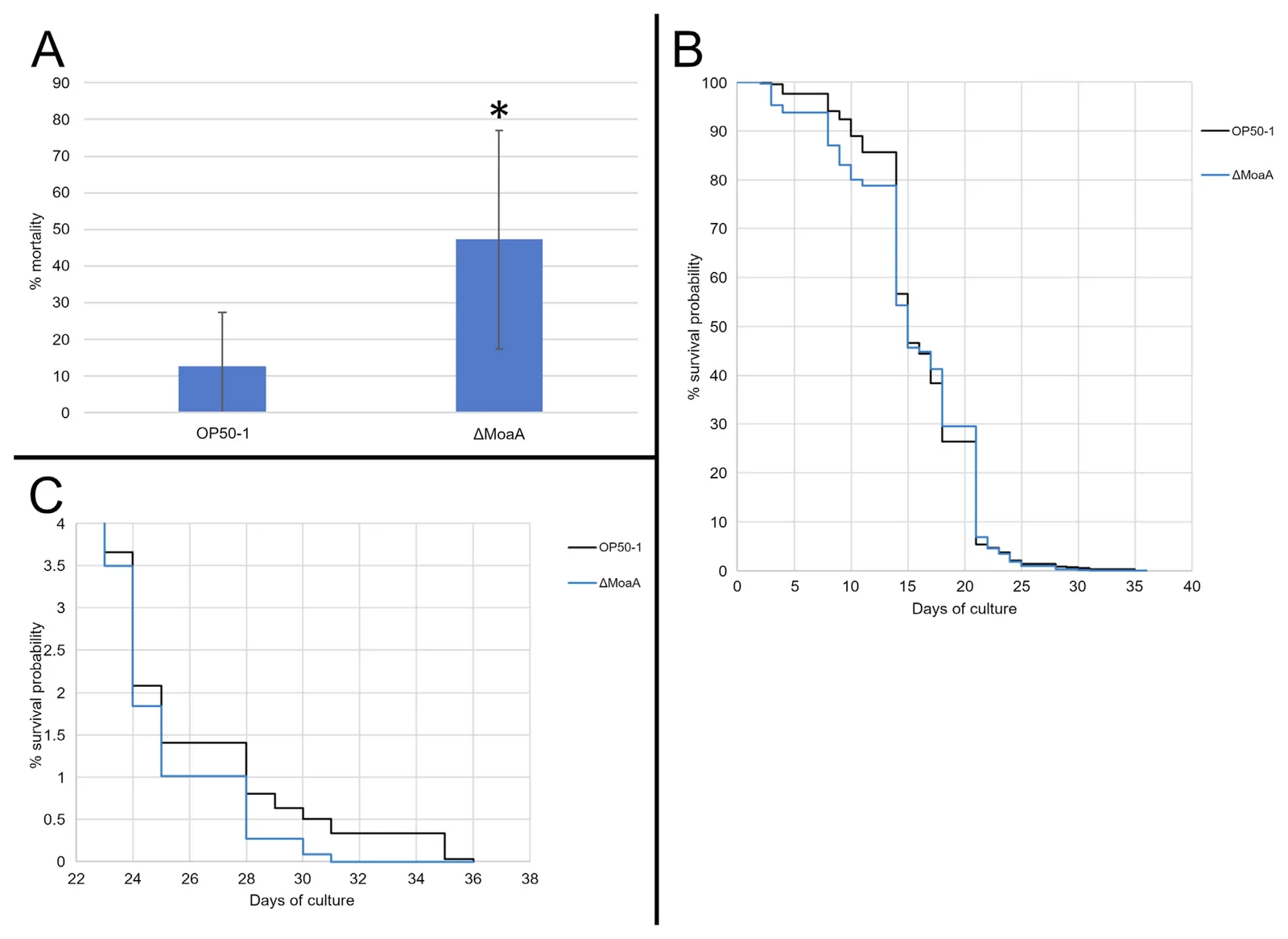
A Molybdenum cofactor (Moco)-restricted diet impairs in vitro cultivation of *Haemonchus contortus* early parasitic stages. (A) Mortality associated with exsheathment using hypochlorite in *H. contortus* L3s that had been fed on a Moco-containing diet (OP50-1) (*N* = 3,566) or a Moco-deficient diet (ΔMoaA) (*N* = 1,676). *P < 0.01. (B) Kaplan-Meier curve of early parasitic *H. contortus* during in vitro culture after free living stages had been fed on a Moco-containing diet (OP50-1) or a Moco-deficient diet (ΔMoaA). (C) Magnification of the Kaplan-Meier curve shown in B for time points after day 25.

Taking advantage of the fact that exogenous supplies of Moco would be limited during axenic in vitro culture, the impact of Moco reserves accumulated during the free-living stages on survival of the early parasitic stages was investigated. *Haemonchus contortus* L3s which had been reared on the Moco-deficient diet (*N* = 1,676) showed 34.7% higher mortality during exsheathment with hypochlorite than the controls (*N* = 3,566) (47.2% versus 12.6%, *P* = 0.008) (Fig. 4A), a nearly four times increase. This initial decimation within 72 h of exsheathment was enough to bias the log rank test used on the Kaplan-Meier curves (Supplementary Fig. S2) resulting in a *P* value of 7.42*10^-17^ meaning a second analysis needed to be conducted which only included mortality that occurred after media addition (Fig. 4B). Within the first 15 days of cultivation, the ΔMoaA reared larvae had 4.9 ± 3.3% higher mortality than the controls (*P* = 0.034). Between 15 and 25 days there were no significant differences in mortality between the two groups, however, after 25 days the remaining ΔMoaA reared early L4s (approximately 2% of the initial population) began to die more rapidly than the controls (*P* = 0.028) (Fig. 4B and enlarged in Fig. 4C). There was a 7 ± 1.6 days difference between 100% mortality being observed for the two groups (*P* = 1.36*10^-7^).

### 3.4 Molybdenum cofactor biosynthesis enzymes have been lost in other Nematoda

With the evidence of innate Moco deficiency in *H. contortus*, which would be unprecedented for a wild multicellular animal, it was examined if this is an exception among nematodes. A BLAST search for molybdenum cofactor synthetic capabilities (genes listed in the boxes of Fig. 1A) was carried out using all nematode species which have an available draft genome, although it was noted that some were assemblies with limited completion (indicated by low Benchmarking Universal Single-Copy Orthologue (BUSCO) scores). Of the 136 species whose genomes were queried (Supplementary Table S2) for *moc-5* and *f49h6.5* orthologs, a further 17 nematode species were found to potentially be unable to synthesize the molybdenum cofactor precursor cyclic pyranopterin monophosphate (cPMP) (Table 1). The absence of these genes in *Teladorsagia circumcincta*, which also has a comprehensive genome assembly (McIntyre et al., 2024; https://ngs.sanger.ac.uk/production/pathogens/sd21/tcircumcincta_genome/reference/) (last accessed 26 March 2024) and is closely related to *H. contortus* and *Haemonchus placei*, gave further confidence that the Orthofinder results were not an artifact caused by missing sequences in the *H. contortus* genome assembly. Of genes for the complex which converts cPMP into molybdopterin (Fig. 1A), *moc-3* orthologs were present in all species queried while the large subunit of molyb-dopterin synthase *moc-4* was absent in the genomes of *Lito-mosoides sigmodontis* and *Panagrolaimus davidi* (Table 1). The gene *moc-6* which encodes the small molybdopterin synthase subunit was less widely conserved on the sequence level with only 25 species having orthologs that had over 50% amino acid identity. This gene also had inconsistent annotation between the protein and DNA databases and the known ortholog from *Brugia malayi, bm10818*, was absent from the returned results. As such BM10818 and the human ortholog MOCS2A were also queried, which uncovered additional orthologs. No orthologs for *moc-6* were found in 11 species, in addition to *H. contortus* (Table 1). Genomes of very few species lacked orthologs of *moc-2* and *moc-1*, which metabolise the last steps in Moco synthesis (Fig. 1A) with only five and three species lacking identifiable orthologs, respectively (Table 1).

**Fig. 5:**
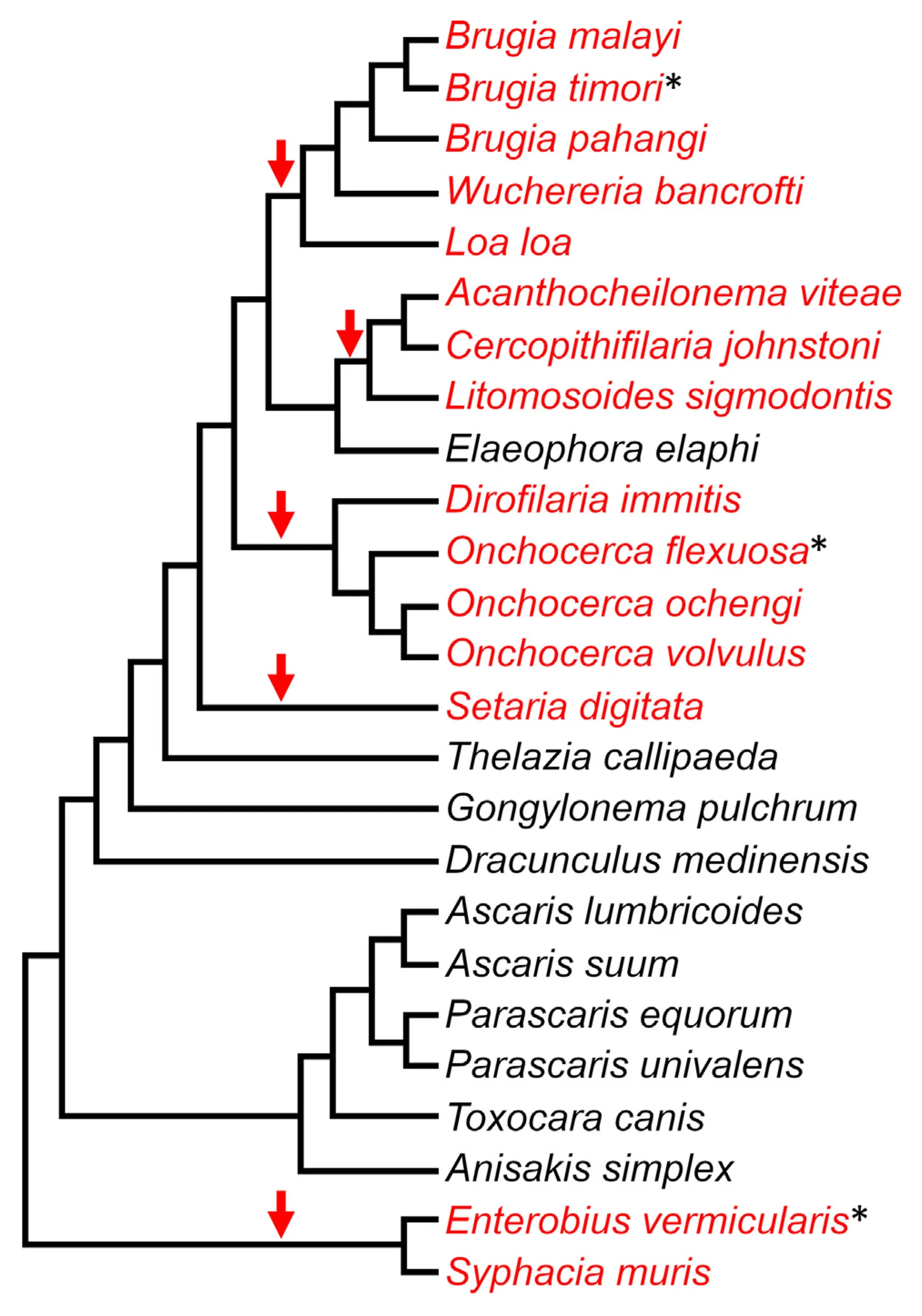
Phylogenetic tree of sequenced genomes from clade III nematodes showing putative Molybdenum cofactor (Moco) synthesis deficiencies. Phylogenetic tree, derived from Ahmed et al. (2022) and McCann et al. (2021), of clade III nematodes with species that lack orthologs of genes for Moco synthesis marked in red. Arrows indicate potential events for Moco synthesis loss. Genome assemblies that had a BUSCO score of < 80% are marked with an asterisk.

The majority of predicted auxotrophs were parasites from the family Onchocercidae (Clade III) but also *Syphacia muris* and *Enterobius vermicularis* from the family Oxyuridae (Clade III) and *Setaria digitata* of the family Setariidae (Clade III). As the 10 other clade III nematode genomes had orthologs for molybdenum cofactor synthesis, including those from the Onchocercidae member *Elaeophora elaphi* and the families Ascaridomorpha, Gongylonematidae and Thelaziidae, there is evidence that synthetic capacity for this cofactor has been lost independently at least five times in this clade (Fig. 5). *Litomosoides sigmodontis* was the only species identified to have lost most of the Moco synthesis pathway. A large reduction in the number of genes for this pathway was also observed in the family Oxyuridae. *Panagrolaimus davidi* was the only free living nematode which may lack a full complement of genes for the Moco synthesis pathway, however the draft genome assembly for this species is largely incomplete (60.7% BUSCO genes missing) (Supplementary Table S2), which is considerably lower than the other four *Panagrolaimus* spp. on WBPS18 (54.7–69.9%). Similarly, as the genome assemblies for *Brugia timori, Ditylenchus destructor, Ditylenchus dipsaci, E. vermicularis, H. placei, Meloidogyne enterolobii, Meloidogyne floridensis, Meloidogyne incognita, Meloidogyne javanica*, and *Onchocerca flexuosa* also have BUSCO scores under 80% (Supplementary Table S2), there is a possibility that missing sequence may result in these species showing false negative outcomes.

## 4 Discussion

The pathway for Moco synthesis is believed to be primordial in origin, highly conserved and present in all multicellular animals (Zhang and Gladyshev, 2008, 2010). Even partial loss of function mutations in this essential pathway are usually associated with highly deleterious phenotypes (Wittle et al., 1999; Lee et al., 2002; Mendel and Hänsch, 2002; Fujii et al., 2016) which puts them under strong negative selective pressure. The results presented here indicate that the phylum *Nematoda*, of which only three representative genomes were available when previous comparative genomics studies were conducted (Zhang and Gladyshev, 2008, 2010), may be an exception, made possible by the capability of nematodes to derive Moco from dietary sources (Warnhoff and Ruvkun, 2019; Warnhoff et al., 2021). The gaps in molybdenum cofactor synthesis in some parasitic nematodes such as *H. contortus* are likely to make Moco an essential nutrient for these species, and may partially explain their inability to adapt to conventional laboratory culture without intermittent access to a host, given the labile nature of the cofactor (Leimkühler et al., 2011; Warnhoff et al., 2021). This also suggests that during in vitro culture fresh Moco would have to be added to media in a protein-bound form, for improved stability (Warnhoff et al., 2021), and would still require frequent replenishment.

**Fig. 6:**
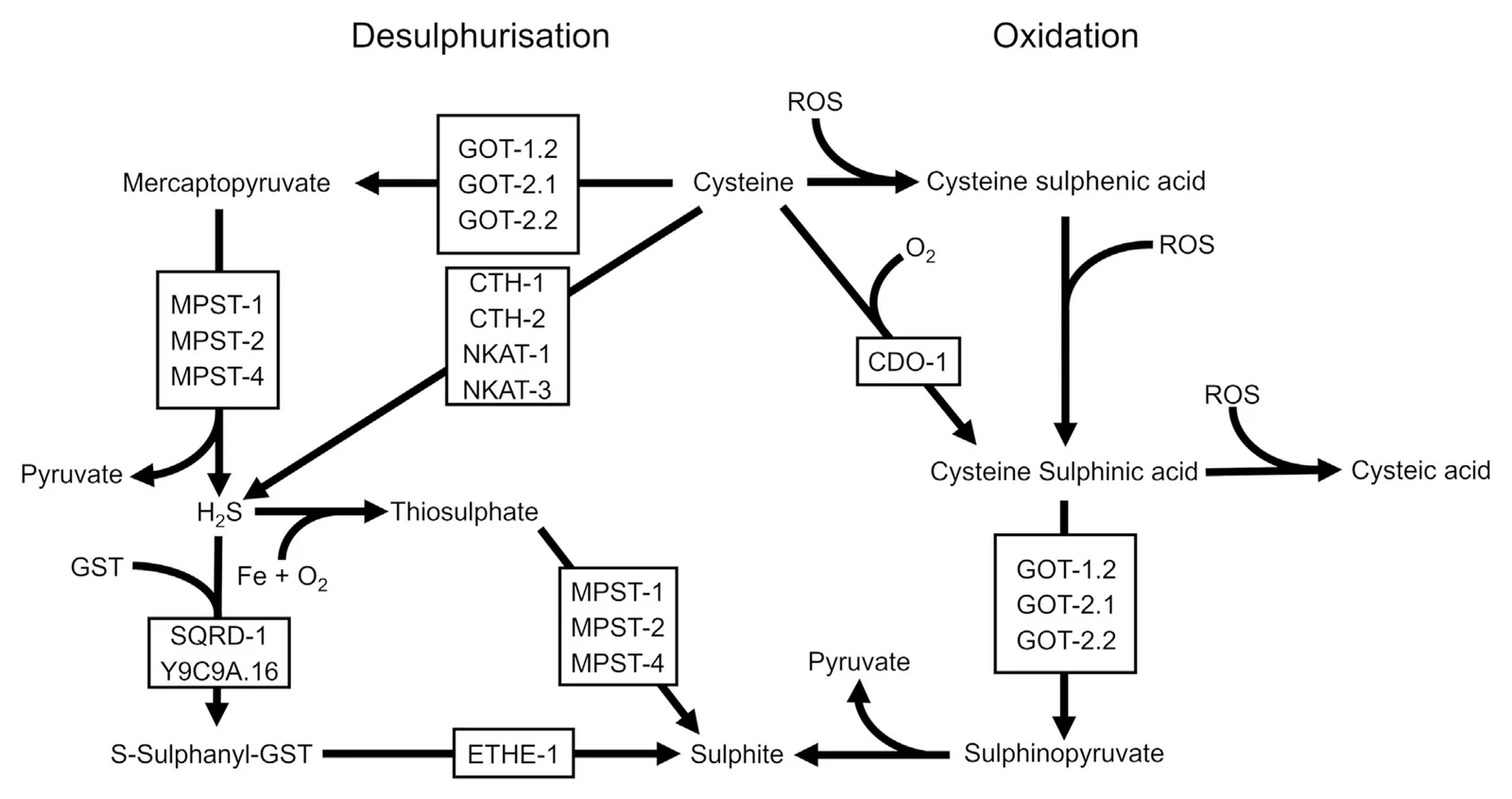
Cysteine catabolism in nematodes. Diagram showing the many routes in nematodes by which cysteine can be catabolised to form sulphite. Boxes indicate enzymes that redundantly metabolise the same step and do not represent subunits. Gene names used are *Caenorhabditis elegans* orthologs. ROS, reactive oxygen species.

Metabolic processes that are dependent on Moco may also explain previous observations made during attempts to optimise *H. contortus* culture. Methods used for liver extract preparation had an important influence on the length of time cultures of the parasitic stages could be maintained (Silverman, 1959). An explanation for this is that in addition to being a good source of vitamin B_12_ and sterols (https://fdc.nal.usda.gov/fdc-app.html#/food-details/169451/nutrients) (last accessed 26 March 2024), the mammalian liver is enriched in Moco and Moco-containing enzymes (Richert and Westerfeld, 1951). Thermally treated extracts would have denatured the thermolabile Moco while differences in fractionation may have caused enrichment or depletion of Moco in the extracts. The addition of cysteine to media formulations was observed to be initially beneficial but became detrimental after prolonged exposure (Schultz, 1967; Mapes, 1970), while of various cell culture media tested, Waymouth’s, which contains free cysteine, was found to be toxic to *Haemonchus* (Hansen et al., 1966). This likely reflects that cysteine catabolism (Fig. 6) generates sulphite which needs to be metabolised by the Moco-dependent enzyme sulphite oxidase (Wyse et al., 2019). During Moco deficiency there is a loss in sulphite oxidase activity, resulting in toxicity through non-enzymatic reactions of sulphite which reduces disulphide bonds in proteins and generates reactive oxygen species and reactive sulphur species (Fig. 1B) (Laggner et al., 2005; Wyse et al., 2019; Maiti, 2022). The importance of controlling excess sulphite may also explain the improved development of *H. contortus* when cultivated under hypoxia as opposed to normoxia (Schultz, 1967), as the reduction in molecular oxygen would slow production of both sulphite by cysteine oxidation pathways (see Fig. 6) and sulphite-derived reactive sulphur species (Maiti, 2022).

The generation of reactive oxidative species by sulphite indicates that *H. contortus* may undergo oxidative stress during Moco depletion which would cause increased susceptibility to other sources of reactive oxygen species such as hypochlorite (Cheshchevik et al., 2021), as occurred following the exsheathment protocol. Interestingly, the bioinformatics results showed that *H. contortus* has also lost the orthologs of *nkat-1* and *nkat-3* (Supplementary Table S1). These are kynurenine amino transferase I/glutamine transaminase K enzymes, so may also have cysteine-S-conjugate beta-lyase activity (Cooper et al., 2011). Loss of these genes would reduce the quantity of sulphite being generated by cysteine desulphurisation catabolic pathways (see Fig. 6) and may provide a protective effect against Moco depletion, similar to that observed in Moco-deficient *C. elegans* mutants which also have a cystathionine gamma-lyase (*cth-2*) or cysteine dioxygenase (*cdo-1*) mutation (Warnhoff and Ruvkun, 2019). Copper plays an important role in catalysing the formation of sulphite-derived oxidative species (Laggner et al., 2005). Notably, *H. contortus* lacks orthologs for the *mtl-1* and *mtl-2* metallothionein genes (Supplementary Table S1), which have important roles in metal tolerance (Zeitoun-Ghandour et al., 2011; Ibiam and Grant, 2015; Yang et al., 2016). Together this might explain the high copper sensitivity that has been reported in *H. contortus* and proposed as an alternative control method (Burke and Miller, 2006, 2008; Dolenga et al., 2023).

The phenotypes of *H. contortus* that occurred during sulphite exposure and Moco depletion were very different from those documented for the free-living nematode *C. elegans* (Warnhoff and Ruvkun, 2019; Oliphant et al., 2023). Despite the OP50-1 fed *H. contortus* larvae having similar sulphite oxidase activity to wild type *C. elegans* (this study; Oliphant et al., 2023), there was mortality on sulphite exposure, suggesting that *Haemonchus* has difficulty in tolerating or repairing sulphite-induced damage. Disulphide bond disruption in the collagen matrix of the cuticle by sulphite (Page and Winter, 2003; Wyse et al., 2019) was probably causal for the cuticle damage observed in *H. contortus* adult worms. If *H. contortus* is as susceptible to sulphite under natural conditions, it would be worth investigating if sulphite releasing compounds or formulations could have application for control.

Relative to Moco-deficient *C. elegans* which show immediate developmental arrest at L1 (Warnhoff and Ruvkun, 2019), the delayed developmental arrest at L3 which occurred in *H. contortus* fed a Moco-deficient diet could be explained by increased maternal provisioning of protein-bound Moco in comparison to *C. elegans*. Alternatively the *cdo-1* ortholog of *Haemonchus*, which catabolizes cysteine via an oxidative pathway (see Fig. 6), is only expressed at very low levels during the free-living stages before upregulation during the infective and parasitic stages (Laing et al., 2013). This would mean that less cysteine-derived sulphite is generated during the early developmental stages, making them more tolerant to Moco deficiency. The hypoxia inducible nature of *cdo-1* (Warnhoff et al., 2023) may also explain the differences in mortality observed when *H. contortus* was reared in the larval development assays, which use a thin covering layer of liquid media, in comparison to plates with air exposed agar surfaces.

The high mortality observed in the exsheathed L3/early L4 populations from both dietary regimes during in vitro culture suggests that RPMI with added FCS is suboptimal for prolonged culture, which may be due to the low essential amino acid and vitamin concentrations compared with media used for culture of other nematode species (Jackson, 1962; Tietjen and Lee, 1975; Szewczyk et al., 2003). Potential explanations for the three separate phases of survival observed are: exsheathed L3s are initially non-feeding and dependent on stored Moco reserves which in the Moco-depleted population would cause increased mortality; as the early L4s began to feed on the media they would have been able to scavenge some xanthine oxidase present in the FCS (Cruz et al., 1983) which would have partially relieved the Moco deficiency (Warnhoff et al., 2021), resulting in the period where both populations showed the same survival rates; as the experiment progressed, Moco sources in the media would have depleted by oxidation, once again leaving the L4s to rely on internal reserves which resulted in increased mortality in the population which was initially Moco-depleted.

Moco bound within proteins, such as sulphite oxidase, has a half-life of 3–4 days at physiological temperatures in the presence of oxygen (Ono and Ito, 1982; Warnhoff et al., 2021). This predicted rate of depletion corresponds well with the 5.6 times difference in sulphite oxidase activity observed under the two dietary regimes and the 7 days difference in survival observed during the in vitro culture. The results indicated that Moco deficiency becomes potentially limiting for the prolonged culture required for development to the adult stages of this parasite (Lapage, 1935; Silverman, 1959; Schultz, 1967; Stringfellow, 1986). Interestingly, the time span of 3 days was found to be the frequency at which media for *Haemonchus* needed to be changed to avoid impaired development (Silverman, 1959), suggesting that a continuous supply of fresh Moco may be a key aspect of successful culture.

Synthesis of the Moco precursor cPMP requires purine triphosphates (Fig. 1A), making it energetically costly, and many parasitic nematode species (especially those from clade III) depend on their host or endosymbionts to provide purine nucleotides (Coghlan et al., 2019). Given the apparent loss of cPMP synthase subunit orthologs (MOC-5 and F49H6.5) on several independent occasions, followed by subsequent loss of the other insufficient subunit, there is indication that the occupied niches of these parasitic nematodes may favour loss of this synthesis pathway. As there is a ubiquitous prevalence of Moco in host species, avoiding synthesis of cPMP while obtaining downsteam metabolites such as Moco from dietary sources would enable a reallocation of purines, potentially providing a selective advantage.

With the exception of *P. davidi*, all nematode species that had apparently lost Moco synthesis genes, which were not *moc-6*, downstream of cPMP also lacked cPMP synthase. If not artifacts of incomplete genome assemblies, this suggests the loss occurred subsequently, potentially driven by the absence of substrates. Although the molybdopterin synthase small subunit gene, *moc-6*, is necessary for Moco synthesis in *C. elegans* (Snoozy et al., 2022), peptides encoding the small subunit can be derived from transcripts for the large subunit (*moc-4*) in some species (Stallmeyer et al., 1999; Hahnewald et al., 2006; Marelja et al., 2018; Toomey et al., 2018). As a consequence, the lack of a *moc-6* ortholog in *H. contortus*, and potentially some other nematode species, may not necessarily represent an inability to synthesize molybdopterin, however, it would be worth investigating the structure and composition of the molybdopterin synthase complexes in these species.

When considering gene absence, it is important to consider that the genome assembly may be incomplete and lacking data that is present within the organism. Closely related members of Onchocercidae, Oxyuridae and Trichostrongylidae with high BUSCO score genome assemblies (Supplementary Table S2) shared a lack of analogous orthologs for *f49h6.5, moc-1, moc-2, moc-5* and *moc-6*, lending support to a potential absence in the species with lower quality genome assemblies, such as *B. timori*, which appears to additionally lack *moc-2*. Nevertheless, two other *Brugia* spp. contain *moc-2* orthologs, meaning further work should be performed on *B. timori* to confirm the absence of this gene. Diversity between species should also be considered when looking for the presence or absence of genes in an assembly. The presence of *moc-6* orthologs in other members of Tylenchoidea, combined with the low homology observed for this gene, means the existence of orthologs which are too divergent to be correctly identified is a possibility for this superfamily.

In conclusion, this study has found that *H. contortus* has naturally occurring Moco deficiency and that Moco is a necessary nutrient for prolonging culture. This means the addition of Moco-containing proteins such as nitrate reductase to media may be necessary for some nematode species. The mechanism(s) nematodes use to import dietary Moco is currently unknown but the data suggests it has an essential function in *H. contortus* and potentially other parasitic nematode species. As this is a feature of nematode biology that is absent from host species, there is a future potential to selectively inhibit Moco uptake as a novel alternative method of control.

## Supplementary Material

Supplementary data to this article can be found online at https://doi.org/10.1016/j.ijpara.2024.11.004.

Supplementary Files

## Figures and Tables

**Table 1:** Nematode species in which Moco synthesis gene orthologs were not found.

Clade	Family	Species	*f49h6.5*	*moc-1*	*moc-2*	*moc-3*	*moc-4*	*moc-5*	*moc-6*
Clade III	Onchocercidae	*Acanthocheilonema viteae*	***X***					**X**	
		*Brugia malayi*	***X***					**X**	
		*Brugia pahangi*	**X**					**X**	
		*Brugia timori*	***X***		**X**			**X**	
		*Cercopithifilaria johnstoni*	**X**					**X**	
		*Dirofilaria immitis*	**X**					**X**	
		*Litomosoides sigmodontis*	**X**	**X**	**X**		**X**	**X**	**X**
		*Loa loa*	**X**					**X**	
		*Onchocerca flexuosa*	**X**					**X**	
		*Onchocerca ochengi*	**X**					**X**	
		*Onchocerca volvulus*	**X**					**X**	
		*Wuchereria bancrofti*	**X**					**X**	
	Setariidae	*Setaria digitata*	**X**					**X**	
	Oxyuridae	*Enterobius vermicularis*	**X**	**X**	**X**			**X**	**X**
		*Syphacia muris*	**X**	**X**	**X**			**X**	**X**
Clade IV	Anguinidae	*Ditylenchus destructor*							**X**
		*Ditylenchus dipsaci*							**X**
	Heteroderidae	*Meloidogyne enterolobii*							**X**
		*Meloidogyne floridensis*							**X**
		*Meloidogyne incognita*							**X**
		*Meloidogyne javanica*							**X**
	Panagrolaimidae	*Panagrolaimus davidi*			**X**		**X**		
Clade V	Trichostrongylidae	*Haemonchus contortus*	**X**					**X**	**X**
		*Haemonchus placei*	**X**					**X**	**X**
		*Teladorsagia circumcincta*	**X**					**X**	**X**

X, no ortholog found using BLAST.
