# Supplementary material for: The parasitic nematode Haemonchus contortus lacks molybdenum cofactor synthesis, leading to sulphite sensitivity and lethality in vitro
Source: Int J Parasitol. Author manuscript; Available in PMC 2026 Sep 4. (PMC7619442; doi:10.1016/j.ijpara.2024.11.004)
Supplement: Supplementary file 1 [file EMS218097-supplement-Supplementary_Files.zip › 1-s2.0-S0020751924002078-mmc3.docx]

**Supplementary Table S2.** Genomes queried using BLAST for Moco synthesis pathway genes. BUSCO scores are from WormBase ParaSite (<https://parasite.wormbase.org/species.html>) (last accessed 26 March 2024) and represent 982 Nematoda-specific BUSCO genes.

| **Clade** | **Species Name** | **Assembly (BioProject ID)** | **BUSCO Score** |
| --- | --- | --- | --- |
| Clade I | *Romanomermis culicivorax* | nRc.2.0 (PRJEB1358) | C: 50.3; F: 9.4; M: 40.3 |
|  | *Soboliphyme baturini* | S_baturini_Dall_Island_0011_upd (PRJEB516) | C: 39.8; F: 12.4; M: 47.8 |
|  | *Trichinella britovi* | T3_ISS120_r1.0 (PRJNA257433) | C: 89.6; F: 7; M: 3.4 |
|  | *Trichinella murrelli* | T5_ISS417_r1.0 (PRJNA257433) | C: 88.7; F: 7.5; M: 3.8 |
|  | *Trichinella nativa* | T2_ISS10_r1.0 (PRJNA257433)  T_nativa_rf_gd (PRJNA179527) | C: 90; F: 6.9; M: 3.1  C: 84.6; F: 9.4; M: 6 |
|  | *Trichinella nelsoni* | T7_ISS37_r1.0 (PRJNA257433) | C: 89.2; F: 7.7; M: 3.1 |
|  | *Trichinella papuae* | T10_ISS1980_r1.0 (PRJNA257433) | C: 88.7; F: 7.2; M: 4.1 |
|  | *Trichinella patagoniensis* | T12_ISS2496_r1.0 (PRJNA257433) | C: 89.8; F: 7; M: 3.2 |
|  | *Trichinella pseudospiralis* | T4_ISS13_r1.0 (PRJNA257433)  T4_ISS141_r1.0 (PRJNA257433)  T4_ISS176_r1.0 (PRJNA257433)  T4_ISS470_r1.0 (PRJNA257433)  T4_ISS588_r1.0 (PRJNA257433) | C: 88; F: 8.5; M: 3.5  C: 85.2; F: 9.7; M: 5.1  C: 87.9; F: 8.4; M: 3.7  C: 87.7; F: 8.7; M: 3.6  C: 87.7; F: 8.6; M: 3.7 |
|  | *Trichinella spiralis* | Trichinella_spiralis-3.7.1 (PRJNA12603)  T1_ISS3_r1.0 (PRJNA257433) | C: 89.3; F: 7.1; M: 3.6  C: 89.7; F: 7.1; M: 3.2 |
|  | *Trichinella sp. T6* | T6_ISS34_r1.0 (PRJNA257433) | C: 89.5; F: 7.2; M: 3.3 |
|  | *Trichinella sp. T8* | T8_ISS272_r1.0 (PRJNA257433) | C: 89.6; F: 6.7; M: 3.7 |
|  | *Trichinella sp. T9* | T9_ISS409_r1.0 (PRJNA257433) | C: 89.2; F: 7; M: 3.8 |
|  | *Trichinella zimbabwensis* | T11_ISS1029_r1.0 (PRJNA257433) | C: 87.5; F: 9; M: 3.5 |
|  | *Trichuris muris* | TMUE3.0 (PRJEB126) | C: 69.9; F: 10.5; M: 19.6 |
|  | *Trichuris suis* | T_suis_1.0.allpaths (PRJNA179528)  Tsuis_adult_male_v1.0 (PRJNA208415)  Tsuis_adult_female_1.0 (PRJNA208416) | C: 70.4; F: 10.5; M: 19.1  C: 70.7; F: 10.5; M: 18.8  C: 70.2; F: 11.1; M: 18.7 |
|  | *Trichuris trichiura* | TTRE3.0 (PRJEB535) | C: 69.6; F: 11.2; M: 19.2 |
| Clade III  Clade III | *Acanthocheilonema viteae* | ASM90053725v1 (PRJEB1697) | C: 90.5; F: 7; M: 2.5 |
|  | *Anisakis simplex* | A_simplex_0011_upd (PRJEB496) | C: 63.9; F: 14; M: 22.1 |
|  | *Ascaris lumbricoides* | A_lumbricoides_Ecuador_v1_5_4 (PRJEB4950) | C: 82.5; F: 8.7; M: 8.8 |
|  | *Ascaris suum* | ASM18702v3 (PRJNA62057)  AscSuum_1.0_submitted (PRJNA80881) | C: 89.5; F: 7.2; M: 3.3  C: 89.8; F: 6.3; M: 3.9 |
|  | *Brugia malayi* | Bmal-4.0 (PRJNA10729) | C: 96.8; F: 1.8; M: 1.4 |
|  | *Brugia pahangi* | B_pahangi_Glasgow_0011_upd (PRJEB497) | C: 89.7; F: 6.6; M: 3.7 |
|  | *Brugia timori* | B_timori_Indonesia_v1_0_4_001_upd (PRJEB4663) | C: 53.3; F: 21.1; M: 25.6 |
|  | *Cercopithifilaria johnstoni* | SD210906 (PRJEB47283) | C: 94.4; F: 4.2; M: 1.4 |
|  | *Dirofilaria immitis* | nDi.2.2 (PRJEB1797)  ICBAS_JMDir_1.0 (PRJNA723804) | C: 91.8; F: 4.5; M: 3.7  C: 76.8; F: 16.4; M: 6.8 |
|  | *Dracunculus medinensis* | D_medinensis_Ghana_v2_0_4 (PRJEB500) | C: 85.6; F: 8.7; M: 5.7 |
|  | *Elaeophora elaphi* | E_elaphi_v1_0_4 (PRJEB502) | C: 87.3; F: 6.2; M: 6.5 |
|  | *Enterobius vermicularis* | E_vermicularis_Canary_Islands_upd (PRJEB503) | C: 79.4; F: 10.2; M: 10.4 |
|  | *Gongylonema pulchrum* | G_pulchrum_Hokkaido_0011_upd (PRJEB505) | C: 31.9; F: 17.2; M: 50.9 |
|  | *Litomosoides sigmodontis* | ASM90053727v1 (PRJEB3075) | C: 91.6; F: 5.5; M: 2.9 |
|  | *Loa loa* | LLoa-hgap-1 (PRJNA246086)  Loa_loa_V3 (PRJNA37757) | C: 97.5; F: 2; M: 0.5  C: 96; F: 3.7; M: 0.3 |
|  | *Onchocerca flexuosa* | O_flexuosa_Cordoba_0011_upd (PRJEB512)  O_flexuosa_1.0.allpaths.pg.lrna (PRJNA230512) | C: 47.3; F: 21.7; M: 31  C: 72.4; F: 4.5; M: 23.1 |
|  | *Onchocerca ochengi* | O_ochengi_Ngaoundere (PRJEB1204)  ASM90053720v1 (PRJEB1465) | C: 85.6; F: 9.5; M: 4.9  C: 86.4; F: 9.5; M: 4.3 |
|  | *Onchocerca volvulus* | ASM49940v2 (PRJEB513) | C: 97.6; F: 1.7; M: 0.7 |
|  | *Parascaris equorum* | P_equorum_v1_0_4 (PRJEB514) | C: 5.1; F: 3.9; M: 91 |
|  | *Parascaris univalens* | ASM225920v1 (PRJNA386823) | C: 91; F: 5.8; M: 3.2 |
|  | *Setaria digitata* | ASM364038v1 (PRJNA479729) | C: 94.6; F: 4.1; M: 1.3 |
|  | *Syphacia muris* | S_muris_Valencia_v1_0_4 (PRJEB524) | C: 84.4; F: 7.2; M: 8.4 |
|  | *Thelazia callipaeda* | T_callipaeda_Ticino_0011_upd (PRJEB1205) | C: 88.6; F: 7; M: 4.4 |
|  | *Toxocara canis* | Toxocara_canis_adult_r1.0 (PRJNA248777)  T_canis_Equador_0011_upd (PRJEB533) | C: 87; F: 8; M: 5  C: 73.5; F: 13.4; M: 13.1 |
|  | *Wuchereria bancrofti* | Wb_PNG_Genome_assembly_pt22 (PRJNA275548)  W_bancrofti_Jakarta_0011_upd (PRJEB536) | C: 93.4; F: 3.9; M: 2.7  C: 74.9; F: 11.5; M: 13.6 |
| Clade IV  Clade IV  Clade IV | *Acrobeloides nanus* | v1 (PRJEB26554) | C: 80.4; F: 8.8; M: 10.8 |
|  | *Aphelenchoides besseyi* | AORJ.fasta (PRJNA834627)  AORT.fasta (PRJNA834627)  APFT.fasta (PRJNA834627)  APVT.fasta (PRJNA834627) | C: 75.8; F: 5.7; M: 18.5  C: 78; F: 5.4; M: 16.6  C: 79.4; F: 4.8; M: 15.8  C: 75.8; F: 5.5; M: 18.7 |
|  | *Aphelenchoides bicaudatus* | Abicaudatus.fasta (PRJNA834627) | C: 73.7; F: 8.7; M: 17.6 |
|  | *Aphelenchoides fujianensis* | Afujianensis.fasta (PRJNA834627) | C: 72.8; F: 9.1; M: 18.1 |
|  | *Bursaphelenchus okinawaensis* | BOKI2 (PRJEB40023) | C: 79.2; F:6.4; M: 14.4 |
|  | *Bursaphelenchus xylophilus* | ASM23113v1_submitted (PRJEA64437)  BXYJ5 (PRJEB40022) | C: 75.8; F: 6.1; M: 18.1  C: 77.7; F: 5.6; M: 16.7 |
|  | *Ditylenchus destructor* | ASM157970v1 (PRJNA312427) | C: 76.2; F: 7.9; M: 15.9 |
|  | *Ditylenchus dipsaci* | D.dipsaci.v1.0 (PRJNA498219) | C: 54.7; F: 13.5; M: 31.8 |
|  | *Globodera pallida* | GPAL001 (PRJEB123)  SA_Gpal_Newton (PRJNA702104)  D383_Gpal_1.0 (PRJNA764088) | C: 43.9; F: 9.2; M: 46.9  C: 59.5; F: 9.7; M: 30.9  C: 54.7; F: 10.6; M: 34.7 |
|  | *Globodera rostochiensis* | WUR_GloGros_L19 (PRJNA695196)  WUR_GloGros_L22 (PRJNA695196)  nGr (PRJEB13504) | C: 41.2; F: 9.4; M: 49.4  C: 59.3; F: 9.9; M: 30.8  C: 58.9; F: 10.3; M: 30.8 |
|  | *Halicephalobus mephisto* | SWDT01000000 (PRJNA528747) | C: 81.4; F: 7.2; M: 11.4 |
|  | *Halicephalobus sp. NKZ332* | ASM976126v1 (PRJNA555616) | C: 82; F: 6.2; M: 11.8 |
|  | *Heterodera glycines* | ASM414822v2 (PRJNA381081) | C: 55.2; F: 10.4; M: 34.4 |
|  | *Heterodera schachtii* | Hsch_Bonn_V1.0 (PRJNA722882)  WUR_Hsch_IRS_1.0 (PRJNA767548) | C: 57.2; F: 11.2; M: 31.6  C: 53.1; F: 11.7; M: 35.2 |
|  | *Meloidogyne arenaria* | ASM90000398v1 (PRJEB8714)  ASM369356v1 (PRJNA340324)  ASM313380v1 (PRJNA438575) | C: 65.1; F: 7.4; M: 28.5  C: 55.7; F: 9.6; M: 34.7  C: 57.9; F: 7.8; M: 34.3 |
|  | *Meloidogyne chitwoodi* | ASM1518303v1 (PRJNA666745) | C: 57.8; F: 9.6; M: 32.6 |
|  | *Meloidogyne enterolobii* | ASM369367v1 (PRJNA340324) | C: 49.7; F: 10.4; M: 39.9 |
|  | *Meloidogyne floridensis* | nMf_1_1 (PRJEB6016)  ASM369360v1 (PRJNA340324) | C: 29.7; F: 10.1; M: 60.2  C: 49.9; F: 8.7; M: 41.4 |
|  | *Meloidogyne graminicola* | Mgraminicola_V1 (PRJNA411966) | C: 40.7; F: 9.5; M: 49.8 |
|  | *Meloidogyne hapla* | Freeze_1 (PRJNA29083) | C: 60.1; F: 9.4; M: 31.5 |
|  | *Meloidogyne incognita* | Meloidogyne_incognita_V3 (PRJEB8714)  ASM369364v1 (PRJNA340324) | C: 61.8; F: 7.8; M: 30.4  C: 52.6; F: 9.1; M: 38.3 |
|  | *Meloidogyne javanica* | ASM90000394v1 (PRJEB8714)  ASM369362v1 (PRJNA340324) | C: 61.1; F: 7.5; M: 31.4  C: 57.7; F: 9.8; M: 32.5 |
|  | *Panagrellus redivivus* | Pred3 (PRJNA186477) | C: 82.1; F: 6.1; M: 11.8 |
|  | *Panagrolaimus davidi* | PDA_v2 (PRJEB32708) | C: 30; F: 9.3; M: 60.7 |
|  | *Panagrolaimus sp. ES5* | ES5_v2 (PRJEB32708) | C: 67.4; F: 10.1; M: 22.5 |
|  | *Panagrolaimus sp. JU765* | JU765_v2 (PRJEB32708) | C: 74.8; F: 8.8; M: 16.4 |
|  | *Panagrolaimus sp. 1159* | PS1159_v2 (PRJEB32708) | C: 74.7; F: 8.7; M: 16.6 |
|  | *Panagrolaimus superbus* | PSU_v2 (PRJEB32708) | C: 69.7; F: 11.1; M: 19.2 |
|  | *Parastrongyloides trichosuri* | P_trichosuri_KNP (PRJEB515) | C: 78.1; F: 7.5; M: 14.4 |
|  | *Rhabditophanes sp. KR3021* | Rhabditophanes_sp_KR3021 (PRJEB1297) | C: 74.1; F: 7; M: 18.9 |
|  | *Steinernema carpocapsae* | ASM75764v3 (PRJNA202318)  S_carpo_v1_submitted (PRJNA202318) | C: 87.2; F: 7.1; M: 5.7  C: 89.4; F: 6.3; M: 4.3 |
|  | *Steinernema feltiae* | S_felt_v1_submitted (PRJNA204661)  S_feltiae_NW_1.0 (PRJNA353610) | C: 86.7; F: 6.2; M: 7.1  C: 87.3; F: 7.3; M: 5.4 |
|  | *Steinernema glaseri* | S_glas_v1_submitted (PRJNA204943) | C: 85.6; F: 6.8; M: 7.6 |
|  | *Steinernema monticolum* | S_monti_v1_submitted (PRJNA205067) | C: 79.9; F: 9.4; M: 10.7 |
|  | *Steinernema scapterisci* | S_scapt_v1_submitted (PRJNA204942) | C: 88.4; F: 6.8; M: 4.8 |
|  | *Strongyloides papillosus* | S_papillosus_LIN_v2_1_4 (PRJEB525) | C: 77.2; F: 7; M: 16.8 |
|  | *Strongyloides ratti* | S_ratti_ED321_v5_0_4 (PRJEB125) | C: 78.1; F: 7.2; M: 14.7 |
|  | *Strongyloides stercoralis* | S_stercoralis_PV0001_v2_0_4 (PRJEB528) | C: 78.5; F: 6.4; M: 15.1 |
|  | *Strongyloides venezuelensis* | S_venezuelensis_HH1 (PRJEB530) | C: 74; F: 7.1; M: 18.9 |
| Clade V  Clade V  Clade V | *Ancylostoma caninum* | A_caninum_9.3.2.ec.cg.pg (PRJNA72585) | C: 84.3; F: 8.7; M: 7 |
|  | *Ancylostoma ceylanicum* | Acey_2013.11.30.genDNA (PRJNA231479)  A_ceylanicum1.3.ec.cg.pg (PRJNA72583) | C: 90.1; F: 6.2; M: 3.7  C: 87.2; F: 7.5; M: 5.3 |
|  | *Ancylostoma duodenale* | A_duodenale_2.2.ec.cg.pg (PRJNA72581) | C: 62.9; F: 15.7; M: 21.4 |
|  | *Angiostrongylus cantonensis* | ACgenome_NGS_v2 (PRJNA350391)  A_cantonensis_Taipei_v1_5_4 (PRJEB493) | C: 83.6; F: 8.4; M: 8  C: 67.8; F: 11.3; M: 20.9 |
|  | *Angiostrongylus costaricensis* | A_costaricensis_Costa_Rica_0011_upd (PRJEB494) | C: 83.1; F: 9.4; M: 7.5 |
|  | *Angiostrongylus vasorum* | UZH_Avas_1.0 (PRJNA663250) | C: 81.9; F: 10; M: 8.1 |
|  | *Caenorhabditis angaria* | ps1010rel4 (PRJNA51225) | C: 77.7; F: 10.3; M: 12 |
|  | *Caenorhabditis auriculariae* | CAUJ (PRJEB40642) | C: 90; F: 5.7; M: 4.3 |
|  | *Caenorhabditis becei* | CBECE_v1 (PRJEB28243) | C: 97.6; F: 1.4; M: 1 |
|  | *Caenorhabditis bovis* | CBOVIS_v1 (PRJEB34497) | C: 94.2; F: 4.6; M: 1.2 |
|  | *Caenorhabditis brenneri* | C_brenneri-6.0.1b (PRJNA20035) | C: 96.6; F: 2.1; M: 1.3 |
|  | *Caenorhabditis briggsae* | CB4 (PRJNA10731)  ASM2149197v1 (PRJNA784955)  ASM2245388v1 (PRJNA784955) | C: 97.7; F: 1.5; M: 0.8  C: 98.5; F: 0.7; M: 0.8  C: 98.5; F: 0.8; M: 0.7 |
|  | *Caenorhabditis elegans* | WBcel235 (PRJNA13758) | C: 98.6; F: 0.8; M: 0.6 |
|  | *Caenorhabditis inopinata* | Sp34_v7 (PRJDB5687) | C: 96.6; F: 2.3; M: 1.1 |
|  | *Caenorhabditis japonica* | C_japonica-7.0.1 (PRJNA12591) | C: 92.4; F: 6.1; M: 1.5 |
|  | *Caenorhabditis latens* | CaeLat1.0 (PRJNA248912) | C: 97; F: 2; M: 1 |
|  | *Caenorhabditis nigoni* | nigoni.pc_2016.07.14 (PRJNA384657) | C: 98.6; F: 1.1; M: 0.3 |
|  | *Caenorhabditis panamensis* | CPANA_v1 (PRJEB28259) | C: 97.3; F: 1.8; M: 0.9 |
|  | *Caenorhabditis parvicauda* | CPARV_v1 (PRJEB12595) | C: 89.5; F: 5; M: 5.5 |
|  | *Caenorhabditis quiockensis* | CQUIO_v1 (PRJEB11354) | C: 95.9; F: 3.3; M: 0.8 |
|  | *Caenorhabditis remanei* | ASM164373v2 (PRJNA248909)  CaeRem1.0 (PRJNA248911)  C_remanei-15.0.1 (PRJNA53967)  CRPX506 (PRJNA577507) | C: 97.6; F: 2; M: 0.4  C: 97; F: 2.1; M: 0.9  C: 97.3; F: 1.9; M: 0.8  C: 97.8; F: 1.2; M: 1 |
|  | *Caenorhabditis sinica* | 1.0 (PRJNA194557) | C: 93.9; F: 4.7; M: 1.4 |
|  | *Caenorhabditis sulstoni* | CSULS_v1 (PRJEB12601) | C: 97.9; F: 1; M: 1.1 |
|  | *Caenorhabditis tribulationis* | CTRIB_v1 (PRJEB12608) | C: 97.7; F: 1.1; M: 1.2 |
|  | *Caenorhabditis tropicalis* | Caenorhabditis_sp11_JU1373-3.0.1 (PRJNA53597) | C: 97; F: 1.9; M: 1.1 |
|  | *Caenorhabditis uteleia* | CUTEL_v1 (PRJEB12600) | C: 96; F: 3.1; M: 0.9 |
|  | *Caenorhabditis waitukubuli* | CWAIT_v1 (PRJEB12602) | C: 92.5; F: 4.8; M: 2.7 |
|  | *Caenorhabditis zanzibari* | CZANZ_v1 (PRJEB12596) | C: 98.2; F: 1.2; M: 0.6 |
|  | *Cylicostephanus goldi* | C_goldi_Cheshire_0011 (PRJEB498) | C: 12.2; F: 11.9; M: 75.9 |
|  | *Dictyocaulus viviparus* | nDv.1.0 (PRJEB5116)  D_viviparus_9.2.1.ec.pg (PRJNA72587) | C: 71.3; F: 12.9; M: 15.8  C: 82.4; F: 10.8; M: 6.8 |
|  | *Diploscapter coronatus* | ASM220778v1 (PRJDB3143) | C: 91.5; F: 4.6; M: 3.9 |
|  | *Diploscapter pachys* | DipSp1Ass11Ann3 (PRJNA280107) | C: 90.6; F: 5.3; M: 4.1 |
|  | *Haemonchus contortus* | haemonchus_contortus_MHCO3ISE_4.0 (PRJEB506)  Hco_v4_coding_submitted (PRJNA205202) | C: 86.6; F: 6.6; M: 6.8  C: 59.7; F: 10.7; M: 29.6 |
|  | *Haemonchus placei* | H_placei_MHpl1_0011_upd (PRJEB509) | C: 79; F: 10.3; M: 10.7 |
|  | *Heligmosomoides polygyrus* | nHp_v2.0 (PRJEB15396)  H_bakeri_Edinburgh_0011_upd (PRJEB1203) | C: 87.1; F: 7.2; M: 5.7  C: 67.8; F: 10.7; M: 21.5 |
|  | *Heterorhabditis bacteriophora* | Heterorhabditis_bacteriophora-7.0 (PRJNA13977) | C: 87.1; F: 9.6; M: 3.3 |
|  | *Mesorhabditis belari* | M.Belari_Hybrid_genome (PRJEB30104) | C: 79.8; F: 7.7; M: 12.5 |
|  | *Micoletzkya japonica* | Micoletzkya_japonica_genome (PRJEB27334) | C: 86.6; F: 6.8; M: 6.6 |
|  | *Necator americanus* | N__americanus_v1 (PRJNA72135) | C: 76.3; F: 11.3; M: 12.4 |
|  | *Nippostrongylus brasiliensis* | N_brasiliensis_RM07_v1_5_4_0011_upd (PRJEB511) | C: 80.1; F: 12.6; M: 7.3 |
|  | *Oesophagostomum dentatum* | O_dentatum_10.0.ec.cg.pg (PRJNA72579) | C: 51.3; F: 18.7; M: 30 |
|  | *Oscheius tipulae* | Oscheius_tipulae_assembly_v2 (PRJEB15512)  ASM1342590v1 (PRJNA644888) | C: 89.4; F: 3.2; M: 7.4  C: 88.7; F: 3.1; M: 8.2 |
|  | *Parapristionchus giblindavisi* | Parapristionchus_giblindavisi_II_pacbio (PRJEB53331) | C: 86.2; F: 7.8; M: 6 |
|  | *Pristionchus arcanus* | P._arcanus_genome (PRJEB27334) | C: 92; F: 4.4; M: 3.6 |
|  | *Pristionchus entomophagus* | P._entomophagus_genome (PRJEB27334) | C: 86.8; F: 6.8; M: 6.4 |
|  | *Pristionchus exspectatus* | exspectatus_chromosome (PRJEB46690) | C: 92; F: 4.2; M: 3.8 |
|  | *Pristionchus fissidentatus* | Pristionchus_fissidentatus_genome (PRJEB27334) | C: 89.6; F: 5.6; M: 4.8 |
|  | *Pristionchus japonicus* | Pristionchus_japonicus_genome (PRJEB27334) | C: 89.8; F: 4.8; M: 5.4 |
|  | *Pristionchus maxplancki* | Prisstionchus_maxplancki_genome (PRJEB27334) | C: 90.6; F: 5.5; M: 3.9 |
|  | *Pristionchus mayeri* | Pristionchus_mayeri_genome (PRJEB27334) | C: 86.7; F: 6.8; M: 6.5 |
|  | *Pristionchus pacificus* | El_Paco (PRJNA12644) | C: 93.2; F: 3.1; M: 3.7 |
|  | *Strongylus vulgaris* | S_vulgaris_Kentucky_0011_upd (PRJEB531) | C: 21.7; F: 15.2; M: 63.1 |
|  | *Teladorsagia circumcincta* | teladorsagia_circumcincta_tci2_wsi.v2.4.fa  T_circumcincta.14.0.ec.cg.pg (PRJNA72569) | C: 85.2; F: 0; M: 14.8  C: 71.8; F: 12; M: 16.2 |
| Clade C | *Plectus sambesii* | Psam_v1.0 (PRJNA390260) | C: 75.9; F: 13.3; M: 10.8 |

C, complete; F, fragmented; M, missing/absent.
